# Crystal structure of *trans*-1-{2-[4-(di­methyl­amino)­phen­yl]eth­yl}-4-[2-(pyren-1-yl)eth­yl]cyclo­hexa­ne

**DOI:** 10.1107/S2056989015013729

**Published:** 2015-07-31

**Authors:** Sreevidya Thekku Veedu, Simone Techert

**Affiliations:** a FS–SCS, Deutsches Elecktronen-Synchrotron (DESY), Notkestrasse 85, 22607 Hamburg, Germany; b Max Planck Institute for Biophysical Chemistry, Am Fassberg 11, 37077 Göttingen, Germany

**Keywords:** crystal structure, pyrene, donor acceptor, electron transfer, C—H⋯π inter­actions

## Abstract

The title compound, C_34_H_37_N, is a pyrene derivative in which the pyrene ring system is linked to an ethyl­cyclo­hexane unit which, in turn, carries a [4-(di­methyl­amino)­phen­yl]ethyl substituent in the *para* position. The central cyclo­hexane ring has a chair conformation, with the exocyclic C—C bonds in equatorial orientations. The benzene ring is inclined to the mean plane of the pyrene ring system [maximum deviation = 0.038 (4) Å] by 14.84 (15)°. In the crystal, mol­ecules are linked by C—H⋯π inter­actions, forming chains propagating along [010]. The crystal was refined as a non-merohedral twin [domain ratio = 0.9989 (4):0.0011 (4)].

## Related literature   

For charge transfer in donor–acceptor systems, see: Wasielewski (1992 ▸); Willemse *et al.* (2000 ▸); Thekku Veedu *et al.* (2014*a* ▸). For related structures, see: Thekku Veedu *et al.* (2014*b* ▸); Wang *et al.* (2010 ▸). For the synthesis of the title compound, see: Dewar & Mole (1956 ▸); Norman *et al.* (1958 ▸).
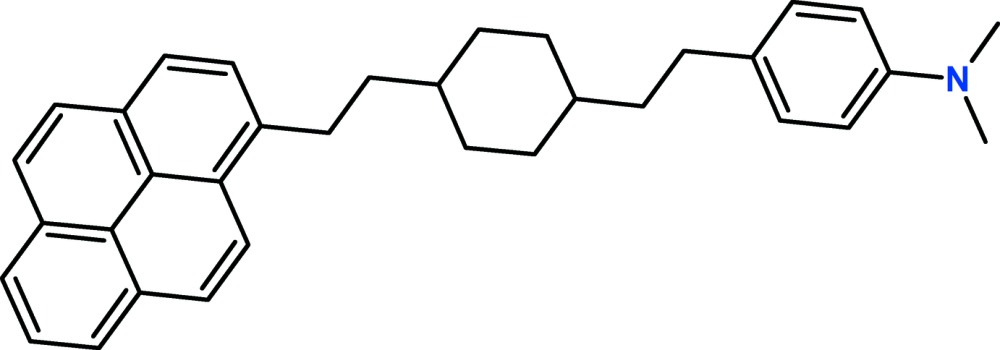


## Experimental   

### Crystal data  

C_34_H_37_N
*M* *_r_* = 459.64
Monoclinic, 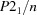

*a* = 7.1927 (4) Å
*b* = 10.4082 (6) Å
*c* = 33.399 (2) Å
β = 91.473 (4)°
*V* = 2499.5 (3) Å^3^
*Z* = 4
Mo *K*α radiation
μ = 0.07 mm^−1^
*T* = 100 K
0.35 × 0.25 × 0.15 mm

### Data collection  

Bruker SMART APEXII DUO diffractometer
Absorption correction: multi-scan (*SADABS*; Bruker, 2012 ▸) *T*_min_ = 0.976, *T*_max_ = 0.990
38082 measured reflections
38082 independent reflections
23477 reflections with *I* > 2σ(*I*)
*R*_int_ = 0.087

### Refinement  

*R*[*F* ^2^ > 2σ(*F* ^2^)] = 0.069
*wR*(*F* ^2^) = 0.195
*S* = 1.07
38082 reflections
320 parameters
H-atom parameters constrained
Δρ_max_ = 0.36 e Å^−3^
Δρ_min_ = −0.32 e Å^−3^

### 

Data collection: *APEX2* (Bruker, 2012 ▸); cell refinement: *SAINT* (Bruker, 2012 ▸); data reduction: *SAINT*; program(s) used to solve structure: *SHELXS2014* (Sheldrick, 2008 ▸); program(s) used to refine structure: *SHELXL2014* (Sheldrick, 2015 ▸); molecular graphics: *SHELXTL* (Sheldrick, 2008 ▸); software used to prepare material for publication: *PLATON* (Spek, 2009 ▸).

## Supplementary Material

Crystal structure: contains datablock(s) global, I. DOI: 10.1107/S2056989015013729/su5171sup1.cif

Structure factors: contains datablock(s) I. DOI: 10.1107/S2056989015013729/su5171Isup2.hkl

Supporting information file. DOI: 10.1107/S2056989015013729/su5171Isup3.cml

**Fig. 1.**
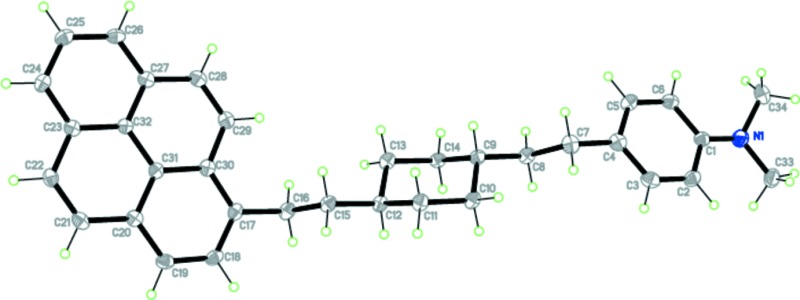
The molecular structure of the title compound, with atom labelling. Displacement ellipsoids are drawn at the 50% probability level.

**Fig. 2.**
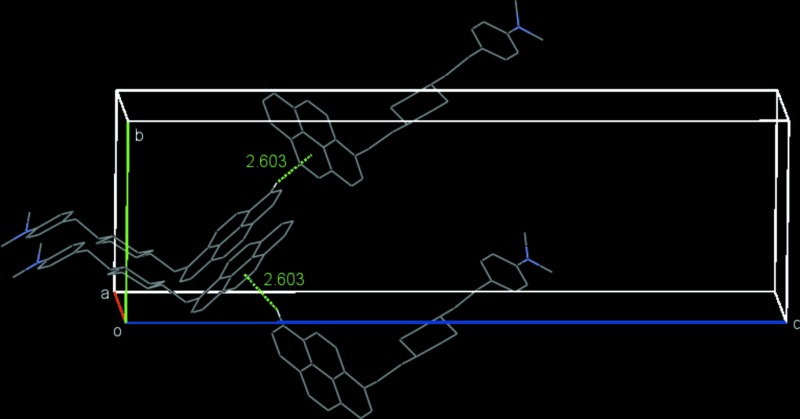
The crystal packing of the title compound, viewed along the a axis. The C—H···π interactions linking the molecules are shown as dashed lines (see Table 1 for details).

. DOI: 10.1107/S2056989015013729/su5171fig1.tif
The mol­ecular structure of the title compound, with atom labelling. Displacement ellipsoids are drawn at the 50% probability level.

a . DOI: 10.1107/S2056989015013729/su5171fig2.tif
The crystal packing of the title compound, viewed along the *a* axis. The C—H⋯π inter­actions linking the mol­ecules are shown as dashed lines (see Table 1 for details).

CCDC reference: 1413890

Additional supporting information:  crystallographic information; 3D view; checkCIF report

## Figures and Tables

**Table 1:** Hydrogen-bond geometry (, ) *Cg*1 is the centroid of the C20C23/C32/C31 ring.

*D*H*A*	*D*H	H*A*	*D* *A*	*D*H*A*
C26H26*Cg*1^i^	0.95	2.60	3.4927(15)	156

Symmetry code: (i) 
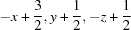
.

## supplementary crystallographic information

### S1. Comment

Electron-transfer reactions are fundamental processes in chemistry and also in biology. Going back to nature, photo-induced electron transfer (PI—ET) is the key step in photosynthesis where light harvesting complexes are functional centers in plants which converts solar energy into chemical energy. In the past decades, to gain more insight into electron transfer processes extensive studies have been carried out on the optical behaviour of systems consisting of donor acceptor groups linked by different bridges (Thekku Veedu *et al.*, 2014a; Wasielewski, 1992; Willemse *et al.*, 2000). These molecules are also ideal systems for studying solvation dynamics and non-linear optical properties. In the title compound (PyDMAD), the electron donor *N,N'*-di­methyl­aniline (DMA) unit is covalently linked to the electron acceptor pyrene by an extended di­ethyl­cyclo­hexane bridge between the donor and acceptor.

The molecular structure of the title pyrene derivative is illustrated in Fig. 1. Pyrene is linked to an ethyl­cyclo­hexane ring which in turn carries a 4-di­methyl­amino­phenyl­ethyl substituent in the *para*-position. The bond lengths and angles are within normal ranges and are comparable to those reported for similar structures (Thekku Veedu *et al.*, 2014b; Wang *et al.*, 2010). The cyclo­hexane ring (C9—C14) has a chair conformation. The benzene ring (C1—C6) is inclined to the mean plane of the pyrene ring system (maximum devation = 0.038 (4) Å for atom C29), by 14.84 (15) °. The various hetero atoms of the di­methyl­amino group are displaced from the benzene ring by 0.078 (4) Å for N1, 0.102 (4) Å for C33, but 0.549 (4) Å for atom C34.

In the crystal, molecules are linked via C—H···π inter­actions forming chains along the *b* axis direction (Table 1 and Fig. 2).

### S2. Synthesis and crystallization

Commercially available 1-amino­pyrene after diazo­tization reaction was coupled with *N,N'*-di­methyl­aniline according to the previously reported procedure (Dewar & Mole 1956; Norman *et al.*, 1958). The crude product was then purified on an aluminium oxide column with a mixture of cyclo­hexane/toluene as eluent and applying HPLC. Plate-like colourless crystals of the title compound were obtained by slow evaporation of a solution in ethyl acetate.

### S3. Refinement

Crystal data, data collection and structure refinement details are summarized in Table 2. The C-bound H-atoms were included in calculated positions and treated as riding atoms: C—H = 0.95 - 1.00 Å with U_iso_(H) = 1.5U_eq_(C) for methyl H atoms and 1.2U_eq_(C) for other H atoms. The crystal was refined as a non-merohedral twin [refined BASF ratio = 0.9989 (4):0.0011 (4)].

### Crystal data

**Table:**

C_34_H_37_N	*F*(000) = 992
*M**_r_* = 459.64	*D*_x_ = 1.221 Mg m^−^^3^
Monoclinic, *P*2_1_/*n*	Mo *K*α radiation, λ = 0.71073 Å
*a* = 7.1927 (4) Å	Cell parameters from 2860 reflections
*b* = 10.4082 (6) Å	θ = 2.5–26.8°
*c* = 33.399 (2) Å	µ = 0.07 mm^−^^1^
β = 91.473 (4)°	*T* = 100 K
*V* = 2499.5 (3) Å^3^	Plate, colorless
*Z* = 4	0.35 × 0.25 × 0.15 mm

### Data collection

**Table:**

Bruker SMART APEXII DUO diffractometer	23477 reflections with *I* > 2σ(*I*)
Radiation source: Micro-focus	*R*_int_ = 0.087
φ and ω scan	θ_max_ = 25.1°, θ_min_ = 2.1°
Absorption correction: multi-scan (*SADABS*; Bruker, 2012)	*h* = −8→8
*T*_min_ = 0.976, *T*_max_ = 0.990	*k* = −12→12
38082 measured reflections	*l* = −39→39
38082 independent reflections	

### Refinement

**Table:**

Refinement on *F*^2^	Secondary atom site location: difference Fourier map
Least-squares matrix: full	Hydrogen site location: inferred from neighbouring sites
*R*[*F*^2^ > 2σ(*F*^2^)] = 0.069	H-atom parameters constrained
*wR*(*F*^2^) = 0.195	*w* = 1/[σ^2^(*F*_o_^2^) + (0.0001*P*)^2^ + 8.046*P*] where *P* = (*F*_o_^2^ + 2*F*_c_^2^)/3
*S* = 1.07	(Δ/σ)_max_ < 0.001
38082 reflections	Δρ_max_ = 0.36 e Å^−^^3^
320 parameters	Δρ_min_ = −0.32 e Å^−^^3^
0 restraints	Extinction correction: *SHELXL2014* (Sheldrick, 2015), Fc^*^=kFc[1+0.001xFc^2^λ^3^/sin(2θ)]^-1/4^
Primary atom site location: structure-invariant direct methods	Extinction coefficient: 0.0031 (4)

### Special details

**Table:**

Geometry. All e.s.d.'s (except the e.s.d. in the dihedral angle between two l.s. planes) are estimated using the full covariance matrix. The cell e.s.d.'s are taken into account individually in the estimation of e.s.d.'s in distances, angles and torsion angles; correlations between e.s.d.'s in cell parameters are only used when they are defined by crystal symmetry. An approximate (isotropic) treatment of cell e.s.d.'s is used for estimating e.s.d.'s involving l.s. planes.
Refinement. Refined as a 2-component twin.

### Fractional atomic coordinates and isotropic or equivalent isotropic displacement parameters (Å^2^)

**Table:**

	*x*	*y*	*z*	*U*_iso_*/*U*_eq_	
C1	−0.7622 (6)	0.4345 (4)	−0.13130 (13)	0.0228 (11)	
C2	−0.6154 (6)	0.3625 (4)	−0.14560 (13)	0.0275 (12)	
H2	−0.6292	0.3197	−0.1706	0.033*	
C3	−0.4497 (7)	0.3522 (4)	−0.12399 (13)	0.0293 (12)	
H3	−0.3522	0.3019	−0.1346	0.035*	
C4	−0.4209 (6)	0.4124 (4)	−0.08757 (13)	0.0248 (12)	
C5	−0.5662 (6)	0.4859 (4)	−0.07374 (13)	0.0275 (12)	
H5	−0.5505	0.5298	−0.0490	0.033*	
C6	−0.7332 (6)	0.4975 (4)	−0.09472 (13)	0.0267 (12)	
H6	−0.8295	0.5488	−0.0842	0.032*	
C7	−0.2415 (6)	0.3977 (4)	−0.06387 (13)	0.0285 (12)	
H7A	−0.2037	0.4827	−0.0532	0.034*	
H7B	−0.1436	0.3684	−0.0821	0.034*	
C8	−0.2532 (6)	0.3027 (4)	−0.02897 (12)	0.0242 (12)	
H8A	−0.3531	0.3312	−0.0112	0.029*	
H8B	−0.2891	0.2175	−0.0398	0.029*	
C9	−0.0743 (6)	0.2881 (4)	−0.00411 (12)	0.0214 (11)	
H9	−0.0325	0.3758	0.0043	0.026*	
C10	0.0814 (6)	0.2270 (4)	−0.02735 (12)	0.0229 (11)	
H10A	0.1087	0.2814	−0.0508	0.027*	
H10B	0.0401	0.1419	−0.0374	0.027*	
C11	0.2574 (6)	0.2107 (4)	−0.00179 (12)	0.0253 (12)	
H11A	0.3071	0.2967	0.0053	0.030*	
H11B	0.3518	0.1660	−0.0177	0.030*	
C12	0.2274 (6)	0.1351 (4)	0.03662 (12)	0.0209 (11)	
H12	0.1892	0.0459	0.0290	0.025*	
C13	0.0694 (6)	0.1953 (4)	0.05960 (12)	0.0245 (12)	
H13A	0.0425	0.1411	0.0831	0.029*	
H13B	0.1087	0.2809	0.0695	0.029*	
C14	−0.1066 (6)	0.2095 (4)	0.03377 (12)	0.0230 (11)	
H14A	−0.2035	0.2519	0.0496	0.028*	
H14B	−0.1528	0.1232	0.0261	0.028*	
C15	0.4070 (6)	0.1263 (4)	0.06140 (12)	0.0243 (12)	
H15A	0.4422	0.2138	0.0704	0.029*	
H15B	0.5065	0.0948	0.0440	0.029*	
C16	0.3996 (6)	0.0392 (4)	0.09817 (12)	0.0260 (12)	
H16A	0.3020	0.0710	0.1160	0.031*	
H16B	0.3644	−0.0487	0.0895	0.031*	
C17	0.5821 (6)	0.0335 (4)	0.12126 (12)	0.0217 (11)	
C18	0.7102 (6)	−0.0601 (4)	0.11161 (13)	0.0251 (12)	
H18	0.6801	−0.1189	0.0907	0.030*	
C19	0.8802 (6)	−0.0707 (4)	0.13140 (12)	0.0237 (12)	
H19	0.9652	−0.1355	0.1237	0.028*	
C20	0.9282 (6)	0.0124 (4)	0.16246 (12)	0.0196 (11)	
C21	1.1005 (6)	0.0024 (4)	0.18479 (12)	0.0233 (11)	
H21	1.1862	−0.0630	0.1780	0.028*	
C22	1.1442 (6)	0.0829 (4)	0.21503 (12)	0.0232 (11)	
H22	1.2603	0.0736	0.2290	0.028*	
C23	1.0191 (6)	0.1825 (4)	0.22666 (12)	0.0195 (11)	
C24	1.0607 (6)	0.2681 (4)	0.25782 (13)	0.0262 (12)	
H24	1.1770	0.2618	0.2718	0.031*	
C25	0.9356 (7)	0.3615 (4)	0.26856 (13)	0.0271 (12)	
H25	0.9661	0.4183	0.2900	0.033*	
C26	0.7664 (6)	0.3732 (4)	0.24839 (13)	0.0254 (12)	
H26	0.6808	0.4373	0.2563	0.031*	
C27	0.7201 (6)	0.2921 (4)	0.21650 (12)	0.0204 (11)	
C28	0.5495 (6)	0.3042 (4)	0.19410 (13)	0.0254 (12)	
H28	0.4646	0.3701	0.2009	0.030*	
C29	0.5058 (6)	0.2241 (4)	0.16343 (13)	0.0236 (11)	
H29	0.3916	0.2359	0.1490	0.028*	
C30	0.6270 (6)	0.1218 (4)	0.15210 (12)	0.0197 (11)	
C31	0.8004 (6)	0.1096 (4)	0.17320 (12)	0.0182 (11)	
C32	0.8462 (6)	0.1942 (4)	0.20562 (12)	0.0183 (11)	
C33	−0.9569 (7)	0.3722 (4)	−0.18918 (13)	0.0396 (14)	
H33A	−0.8768	0.4107	−0.2092	0.059*	
H33B	−1.0870	0.3778	−0.1984	0.059*	
H33C	−0.9228	0.2818	−0.1853	0.059*	
C34	−1.0444 (6)	0.5568 (4)	−0.14819 (14)	0.0340 (13)	
H34A	−1.1253	0.5494	−0.1251	0.051*	
H34B	−1.1207	0.5681	−0.1727	0.051*	
H34C	−0.9619	0.6310	−0.1445	0.051*	
N1	−0.9334 (5)	0.4406 (4)	−0.15151 (11)	0.0309 (11)	

### Atomic displacement parameters (Å^2^)

**Table:**

	*U*^11^	*U*^22^	*U*^33^	*U*^12^	*U*^13^	*U*^23^
C1	0.025 (3)	0.019 (3)	0.025 (3)	−0.002 (2)	0.003 (2)	0.002 (2)
C2	0.031 (3)	0.031 (3)	0.021 (3)	0.000 (3)	0.003 (2)	−0.003 (2)
C3	0.025 (3)	0.030 (3)	0.033 (3)	0.003 (2)	0.011 (2)	0.000 (2)
C4	0.023 (3)	0.025 (3)	0.028 (3)	−0.004 (2)	0.005 (2)	0.003 (2)
C5	0.030 (3)	0.029 (3)	0.024 (3)	−0.005 (3)	0.001 (2)	−0.006 (2)
C6	0.026 (3)	0.025 (3)	0.030 (3)	0.004 (2)	0.005 (2)	−0.004 (2)
C7	0.026 (3)	0.029 (3)	0.031 (3)	−0.005 (2)	0.002 (2)	0.005 (2)
C8	0.021 (3)	0.027 (3)	0.024 (3)	−0.001 (2)	0.003 (2)	−0.003 (2)
C9	0.023 (3)	0.018 (3)	0.024 (3)	−0.006 (2)	0.002 (2)	−0.001 (2)
C10	0.022 (3)	0.029 (3)	0.018 (2)	−0.004 (2)	0.003 (2)	0.001 (2)
C11	0.021 (3)	0.032 (3)	0.023 (3)	0.000 (2)	0.005 (2)	−0.002 (2)
C12	0.020 (3)	0.024 (3)	0.020 (3)	−0.005 (2)	0.003 (2)	0.001 (2)
C13	0.025 (3)	0.026 (3)	0.022 (3)	−0.005 (2)	0.004 (2)	0.001 (2)
C14	0.018 (3)	0.028 (3)	0.023 (3)	−0.002 (2)	0.006 (2)	−0.003 (2)
C15	0.022 (3)	0.028 (3)	0.023 (3)	−0.002 (2)	0.003 (2)	−0.002 (2)
C16	0.026 (3)	0.029 (3)	0.023 (3)	−0.004 (2)	0.000 (2)	0.000 (2)
C17	0.025 (3)	0.023 (3)	0.017 (3)	−0.004 (2)	0.002 (2)	0.003 (2)
C18	0.036 (3)	0.022 (3)	0.017 (3)	−0.004 (2)	0.002 (2)	−0.002 (2)
C19	0.030 (3)	0.020 (3)	0.021 (3)	0.003 (2)	0.007 (2)	0.001 (2)
C20	0.022 (3)	0.019 (3)	0.018 (2)	−0.002 (2)	0.005 (2)	0.004 (2)
C21	0.026 (3)	0.021 (3)	0.024 (3)	0.003 (2)	0.006 (2)	0.007 (2)
C22	0.023 (3)	0.024 (3)	0.023 (3)	0.001 (2)	0.000 (2)	0.006 (2)
C23	0.023 (3)	0.018 (3)	0.017 (3)	−0.002 (2)	0.004 (2)	0.004 (2)
C24	0.027 (3)	0.030 (3)	0.022 (3)	−0.004 (2)	−0.002 (2)	0.001 (2)
C25	0.035 (3)	0.025 (3)	0.022 (3)	−0.005 (3)	0.003 (2)	−0.004 (2)
C26	0.029 (3)	0.021 (3)	0.026 (3)	0.000 (2)	0.007 (2)	−0.003 (2)
C27	0.020 (3)	0.019 (3)	0.022 (3)	−0.001 (2)	0.006 (2)	0.003 (2)
C28	0.024 (3)	0.023 (3)	0.029 (3)	0.002 (2)	0.005 (2)	0.002 (2)
C29	0.022 (3)	0.024 (3)	0.025 (3)	−0.002 (2)	0.002 (2)	0.003 (2)
C30	0.021 (3)	0.021 (3)	0.018 (2)	−0.004 (2)	0.005 (2)	0.004 (2)
C31	0.023 (3)	0.016 (2)	0.015 (2)	−0.003 (2)	0.004 (2)	0.004 (2)
C32	0.020 (3)	0.017 (3)	0.017 (2)	−0.004 (2)	0.004 (2)	0.005 (2)
C33	0.051 (4)	0.035 (3)	0.031 (3)	−0.005 (3)	−0.010 (3)	0.001 (3)
C34	0.029 (3)	0.038 (3)	0.036 (3)	0.001 (3)	0.000 (2)	0.009 (2)
N1	0.030 (2)	0.034 (3)	0.029 (2)	0.003 (2)	−0.005 (2)	−0.0074 (19)

### Geometric parameters (Å, º)

**Table:**

C1—C2	1.389 (6)	C16—H16A	0.9900
C1—N1	1.390 (5)	C16—H16B	0.9900
C1—C6	1.397 (6)	C17—C18	1.385 (6)
C2—C3	1.382 (6)	C17—C30	1.412 (6)
C2—H2	0.9500	C18—C19	1.379 (6)
C3—C4	1.380 (6)	C18—H18	0.9500
C3—H3	0.9500	C19—C20	1.387 (5)
C4—C5	1.384 (6)	C19—H19	0.9500
C4—C7	1.504 (6)	C20—C31	1.419 (6)
C5—C6	1.380 (6)	C20—C21	1.433 (6)
C5—H5	0.9500	C21—C22	1.343 (6)
C6—H6	0.9500	C21—H21	0.9500
C7—C8	1.532 (6)	C22—C23	1.433 (6)
C7—H7A	0.9900	C22—H22	0.9500
C7—H7B	0.9900	C23—C24	1.396 (6)
C8—C9	1.521 (5)	C23—C32	1.418 (6)
C8—H8A	0.9900	C24—C25	1.378 (6)
C8—H8B	0.9900	C24—H24	0.9500
C9—C10	1.518 (6)	C25—C26	1.381 (6)
C9—C14	1.529 (5)	C25—H25	0.9500
C9—H9	1.0000	C26—C27	1.393 (6)
C10—C11	1.518 (5)	C26—H26	0.9500
C10—H10A	0.9900	C27—C32	1.417 (6)
C10—H10B	0.9900	C27—C28	1.426 (6)
C11—C12	1.525 (5)	C28—C29	1.351 (6)
C11—H11A	0.9900	C28—H28	0.9500
C11—H11B	0.9900	C29—C30	1.433 (6)
C12—C15	1.519 (5)	C29—H29	0.9500
C12—C13	1.522 (6)	C30—C31	1.422 (6)
C12—H12	1.0000	C31—C32	1.428 (5)
C13—C14	1.520 (5)	C33—N1	1.452 (5)
C13—H13A	0.9900	C33—H33A	0.9800
C13—H13B	0.9900	C33—H33B	0.9800
C14—H14A	0.9900	C33—H33C	0.9800
C14—H14B	0.9900	C34—N1	1.454 (5)
C15—C16	1.529 (5)	C34—H34A	0.9800
C15—H15A	0.9900	C34—H34B	0.9800
C15—H15B	0.9900	C34—H34C	0.9800
C16—C17	1.507 (6)		
			
C2—C1—N1	122.0 (4)	H15A—C15—H15B	107.5
C2—C1—C6	117.1 (4)	C17—C16—C15	112.7 (4)
N1—C1—C6	120.9 (4)	C17—C16—H16A	109.0
C3—C2—C1	121.0 (4)	C15—C16—H16A	109.0
C3—C2—H2	119.5	C17—C16—H16B	109.0
C1—C2—H2	119.5	C15—C16—H16B	109.0
C4—C3—C2	122.2 (5)	H16A—C16—H16B	107.8
C4—C3—H3	118.9	C18—C17—C30	119.1 (4)
C2—C3—H3	118.9	C18—C17—C16	119.0 (4)
C3—C4—C5	116.6 (4)	C30—C17—C16	121.9 (4)
C3—C4—C7	121.6 (4)	C19—C18—C17	122.1 (4)
C5—C4—C7	121.7 (4)	C19—C18—H18	118.9
C6—C5—C4	122.3 (4)	C17—C18—H18	118.9
C6—C5—H5	118.9	C18—C19—C20	120.6 (4)
C4—C5—H5	118.9	C18—C19—H19	119.7
C5—C6—C1	120.8 (4)	C20—C19—H19	119.7
C5—C6—H6	119.6	C19—C20—C31	118.9 (4)
C1—C6—H6	119.6	C19—C20—C21	122.6 (4)
C4—C7—C8	113.8 (4)	C31—C20—C21	118.5 (4)
C4—C7—H7A	108.8	C22—C21—C20	122.0 (4)
C8—C7—H7A	108.8	C22—C21—H21	119.0
C4—C7—H7B	108.8	C20—C21—H21	119.0
C8—C7—H7B	108.8	C21—C22—C23	121.2 (4)
H7A—C7—H7B	107.7	C21—C22—H22	119.4
C9—C8—C7	114.7 (4)	C23—C22—H22	119.4
C9—C8—H8A	108.6	C24—C23—C32	118.9 (4)
C7—C8—H8A	108.6	C24—C23—C22	122.7 (4)
C9—C8—H8B	108.6	C32—C23—C22	118.5 (4)
C7—C8—H8B	108.6	C25—C24—C23	121.0 (4)
H8A—C8—H8B	107.6	C25—C24—H24	119.5
C10—C9—C8	112.8 (3)	C23—C24—H24	119.5
C10—C9—C14	109.2 (4)	C24—C25—C26	120.5 (4)
C8—C9—C14	111.2 (4)	C24—C25—H25	119.7
C10—C9—H9	107.8	C26—C25—H25	119.7
C8—C9—H9	107.8	C25—C26—C27	120.6 (4)
C14—C9—H9	107.8	C25—C26—H26	119.7
C9—C10—C11	112.0 (3)	C27—C26—H26	119.7
C9—C10—H10A	109.2	C26—C27—C32	119.3 (4)
C11—C10—H10A	109.2	C26—C27—C28	122.2 (4)
C9—C10—H10B	109.2	C32—C27—C28	118.5 (4)
C11—C10—H10B	109.2	C29—C28—C27	121.6 (4)
H10A—C10—H10B	107.9	C29—C28—H28	119.2
C10—C11—C12	113.4 (4)	C27—C28—H28	119.2
C10—C11—H11A	108.9	C28—C29—C30	121.8 (4)
C12—C11—H11A	108.9	C28—C29—H29	119.1
C10—C11—H11B	108.9	C30—C29—H29	119.1
C12—C11—H11B	108.9	C17—C30—C31	119.0 (4)
H11A—C11—H11B	107.7	C17—C30—C29	123.2 (4)
C15—C12—C13	112.7 (4)	C31—C30—C29	117.9 (4)
C15—C12—C11	110.6 (4)	C20—C31—C30	120.3 (4)
C13—C12—C11	109.6 (4)	C20—C31—C32	119.5 (4)
C15—C12—H12	107.9	C30—C31—C32	120.2 (4)
C13—C12—H12	107.9	C27—C32—C23	119.6 (4)
C11—C12—H12	107.9	C27—C32—C31	120.0 (4)
C14—C13—C12	112.0 (3)	C23—C32—C31	120.4 (4)
C14—C13—H13A	109.2	N1—C33—H33A	109.5
C12—C13—H13A	109.2	N1—C33—H33B	109.5
C14—C13—H13B	109.2	H33A—C33—H33B	109.5
C12—C13—H13B	109.2	N1—C33—H33C	109.5
H13A—C13—H13B	107.9	H33A—C33—H33C	109.5
C13—C14—C9	112.3 (4)	H33B—C33—H33C	109.5
C13—C14—H14A	109.1	N1—C34—H34A	109.5
C9—C14—H14A	109.1	N1—C34—H34B	109.5
C13—C14—H14B	109.1	H34A—C34—H34B	109.5
C9—C14—H14B	109.1	N1—C34—H34C	109.5
H14A—C14—H14B	107.9	H34A—C34—H34C	109.5
C12—C15—C16	115.2 (4)	H34B—C34—H34C	109.5
C12—C15—H15A	108.5	C1—N1—C33	118.7 (4)
C16—C15—H15A	108.5	C1—N1—C34	118.8 (4)
C12—C15—H15B	108.5	C33—N1—C34	115.0 (4)
C16—C15—H15B	108.5		
			
N1—C1—C2—C3	−176.5 (4)	C32—C23—C24—C25	0.9 (7)
C6—C1—C2—C3	1.2 (7)	C22—C23—C24—C25	−179.0 (4)
C1—C2—C3—C4	−0.2 (7)	C23—C24—C25—C26	−0.6 (7)
C2—C3—C4—C5	−0.8 (7)	C24—C25—C26—C27	−0.9 (7)
C2—C3—C4—C7	178.4 (4)	C25—C26—C27—C32	1.9 (6)
C3—C4—C5—C6	1.0 (7)	C25—C26—C27—C28	−177.8 (4)
C7—C4—C5—C6	−178.3 (4)	C26—C27—C28—C29	−179.5 (4)
C4—C5—C6—C1	0.0 (7)	C32—C27—C28—C29	0.8 (7)
C2—C1—C6—C5	−1.1 (7)	C27—C28—C29—C30	0.8 (7)
N1—C1—C6—C5	176.7 (4)	C18—C17—C30—C31	−1.9 (6)
C3—C4—C7—C8	−101.6 (5)	C16—C17—C30—C31	178.9 (4)
C5—C4—C7—C8	77.7 (6)	C18—C17—C30—C29	178.2 (4)
C4—C7—C8—C9	−178.9 (4)	C16—C17—C30—C29	−1.0 (6)
C7—C8—C9—C10	−66.3 (5)	C28—C29—C30—C17	177.4 (4)
C7—C8—C9—C14	170.6 (4)	C28—C29—C30—C31	−2.5 (6)
C8—C9—C10—C11	−178.8 (4)	C19—C20—C31—C30	−0.6 (6)
C14—C9—C10—C11	−54.7 (5)	C21—C20—C31—C30	−179.3 (4)
C9—C10—C11—C12	55.3 (5)	C19—C20—C31—C32	178.8 (4)
C10—C11—C12—C15	−178.0 (4)	C21—C20—C31—C32	0.0 (6)
C10—C11—C12—C13	−53.2 (5)	C17—C30—C31—C20	1.9 (6)
C15—C12—C13—C14	177.1 (4)	C29—C30—C31—C20	−178.2 (4)
C11—C12—C13—C14	53.5 (5)	C17—C30—C31—C32	−177.4 (4)
C12—C13—C14—C9	−56.7 (5)	C29—C30—C31—C32	2.5 (6)
C10—C9—C14—C13	55.9 (5)	C26—C27—C32—C23	−1.6 (6)
C8—C9—C14—C13	−179.0 (4)	C28—C27—C32—C23	178.2 (4)
C13—C12—C15—C16	63.9 (5)	C26—C27—C32—C31	179.6 (4)
C11—C12—C15—C16	−173.0 (4)	C28—C27—C32—C31	−0.7 (6)
C12—C15—C16—C17	179.5 (4)	C24—C23—C32—C27	0.2 (6)
C15—C16—C17—C18	−90.4 (5)	C22—C23—C32—C27	−179.9 (4)
C15—C16—C17—C30	88.8 (5)	C24—C23—C32—C31	179.0 (4)
C30—C17—C18—C19	0.6 (7)	C22—C23—C32—C31	−1.0 (6)
C16—C17—C18—C19	179.8 (4)	C20—C31—C32—C27	179.7 (4)
C17—C18—C19—C20	0.8 (7)	C30—C31—C32—C27	−1.0 (6)
C18—C19—C20—C31	−0.8 (6)	C20—C31—C32—C23	0.8 (6)
C18—C19—C20—C21	177.9 (4)	C30—C31—C32—C23	−179.8 (4)
C19—C20—C21—C22	−179.4 (4)	C2—C1—N1—C33	−1.6 (7)
C31—C20—C21—C22	−0.7 (7)	C6—C1—N1—C33	−179.2 (4)
C20—C21—C22—C23	0.5 (7)	C2—C1—N1—C34	−149.8 (4)
C21—C22—C23—C24	−179.7 (4)	C6—C1—N1—C34	32.5 (6)
C21—C22—C23—C32	0.4 (6)		

### Hydrogen-bond geometry (Å, º)

Cg1 is the centroid of the C20–C23/C32/C31 ring.

**Table:**

*D*—H···*A*	*D*—H	H···*A*	*D*···*A*	*D*—H···*A*
C26—H26···*Cg*1^i^	0.95	2.60	3.4927 (15)	156

Symmetry code: (i) −*x*+3/2, *y*+1/2, −*z*+1/2.
