# Supplementary figures and images for: Crystal structure of trans-1-{2-[4-(di­methyl­amino)­phen­yl]eth­yl}-4-[2-(pyren-1-yl)eth­yl]cyclo­hexa­ne
Source: Acta Crystallogr E Crystallogr Commun. 2015 Jul 31;71(Pt 8):o629–30. doi: 10.1107/S2056989015013729 (PMC4650969; doi:10.1107/S2056989015013729)

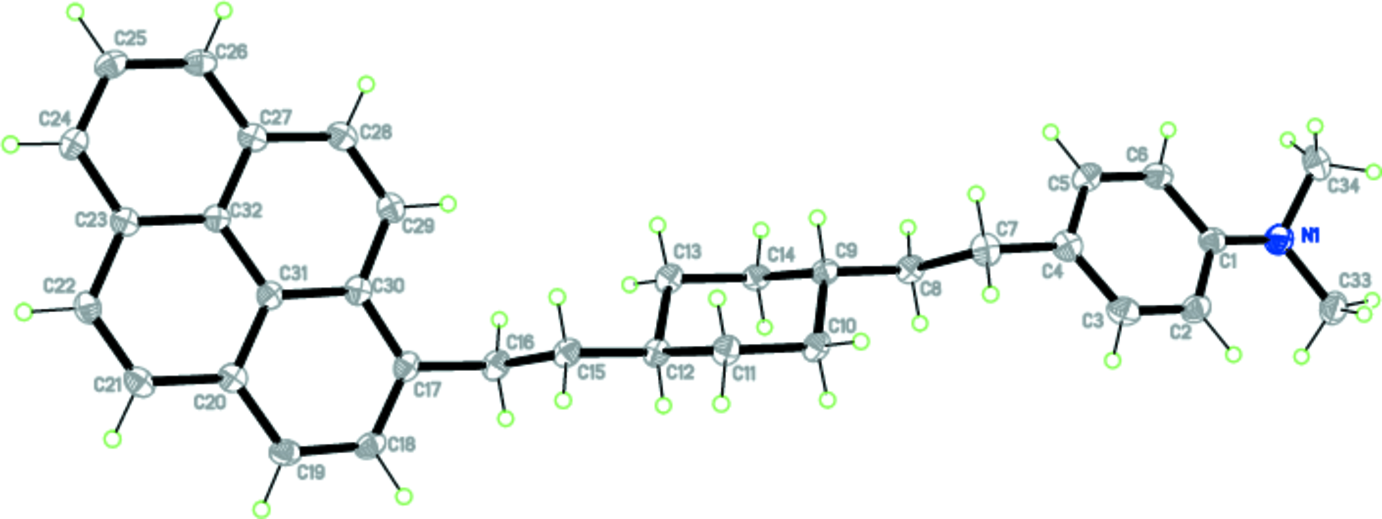

Supplement: Supplementary file 4 [file e-71-0o629-fig1.tif]

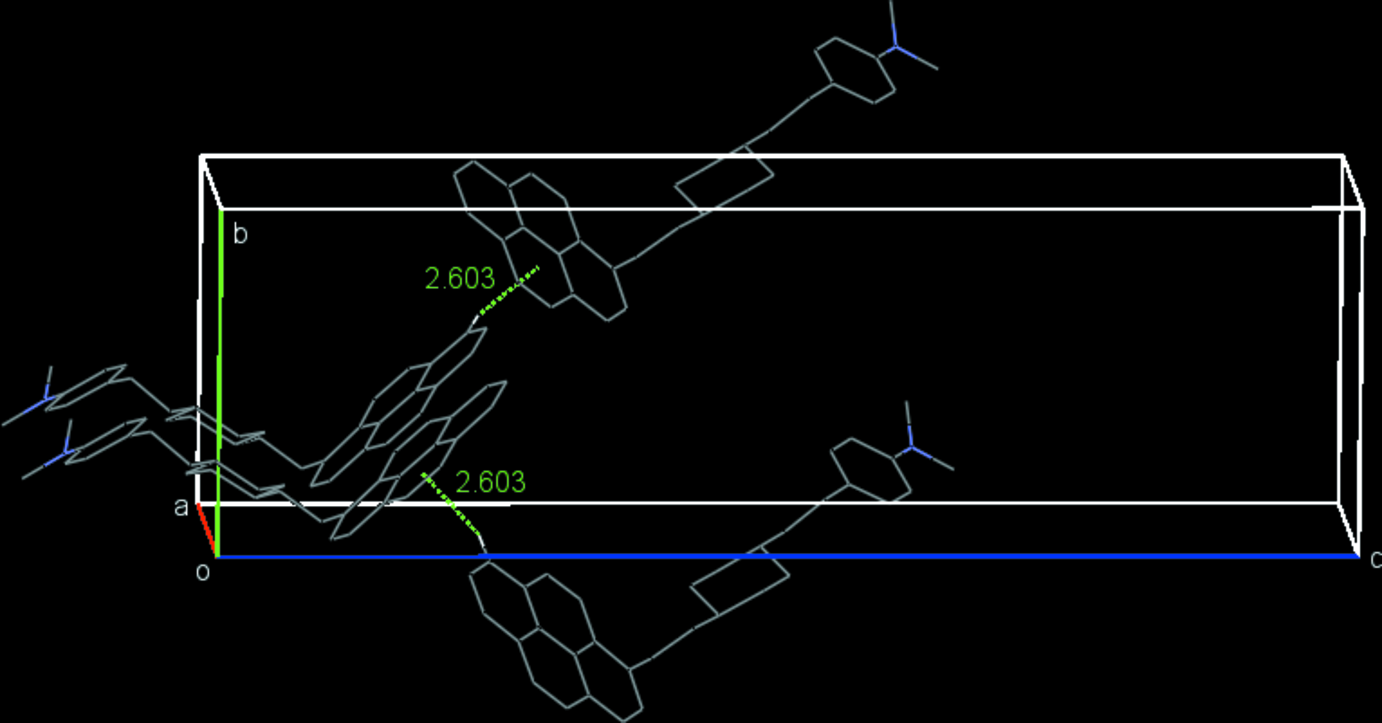

Supplement: Supplementary file 5 [file e-71-0o629-fig2.tif]
